# Persistent repeated measurements by Magnetic Resonance Spectroscopy demonstrate Minimal Hepatic Encephalopathy: a case report

**Published:** 2013-09-25

**Authors:** C Scheau, GA Popa, AE Ghergus, EM Preda, RA Capsa, IG Lupescu

**Affiliations:** Department of Radiology and Medical Imaging, “Fundeni" Clinical Institute, “Carol Davila" University of Medicine and Pharmacy, Bucharest, Romania

**Keywords:** Minimal Hepatic Encephalopathy, Magnetic Resonance Spectroscopy, portosystemic encephalopathy

## Abstract

Minimal Hepatic Encephalopathy (MHE), previously referred to as infraclinical or subclinical is a precursor in the development of clinical hepatic encephalopathy (HE). The demonstration of MHE is done through neuropsychological testing in the absence of clinical evidence of HE, patients showing only a mild cognitive impairment.

Neuropsychological tests employed consist of Repeatable Battery for the Assessment of Neuropsychological Status (RBANS) and portosystemic encephalopathy (PSE) test score. Unfortunately, there are numerous occasions when the tests prove irrelevant: in the situation of inexperienced investigators, the patient’s poor education, vision problems or concurring central nervous system disease, all of which may delay or deviate from the correct diagnosis.

## Introduction

Minimal Hepatic Encephalopathy (MHE), previously referred to as infraclinical or subclinical is a precursor in the development of clinical hepatic encephalopathy (HE). The demonstration of MHE is done through neuropsychological testing in the absence of clinical evidence of HE, patients showing only a mild cognitive impairment [**1**]. 

 Neuropsychological tests employed consist of Repeatable Battery for the Assessment of Neuropsychological Status (RBANS) and portosystemic encephalopathy (PSE) test score [**2**]. Unfortunately, there are numerous occasions when the tests prove irrelevant: in the situation of inexperienced investigators, the patient’s poor education, vision problems or concurring central nervous system disease, all of which may delay or deviate from the correct diagnosis. 

 Magnetic Resonance Imaging can supply the clinician with both morphological and functional information, the latter with the support of Magnetic Resonance Spectroscopy. A series of cerebral metabolites can be assessed non-invasively, bringing useful clinical information [**3**].

## Case presentation

We present the case of a 49-year-old male, with priors of gastro-esophageal reflux disease (GERD), overweight and chronic constipation. The patient is also a chronically alcohol abuser.

 The patient reported to the surgical department of our hospital for the cure of an uncomplicated inguinal hernia. Familiar with the patient’s priors, the surgeon decided to direct the patient to the gastroenterology service to correct his conditions, as each may cause an increased abdominal pressure through Valsalvamaneuver (chronic cough in the case of GERD, and increased defecating efforts related to chronic constipation) or by excessive intra-abdominal volume associating muscular laxity (overweight).

The gastroenterologist noticed slight behavioral anomalies, which he ascribed to a possible hepatic encephalopathy due to a degree of liver failure in the context of long-term alcohol consumption. Blood work examination results at this moment are listed in Table 1 and are consistent with the chronical liver disease due to alcohol abuse. The doctor addresses the patient to the psychologist on call who has difficulty employing the neuropsychological tests required, due to the advanced vision problems of the patient who is unable to complete the forms.

**Table 1:** Blood test results for liver function evaluation

Parameter analyzed	Result	Reference interval /measurement unit
GPT (ALT)	38.00	0 – 45 U/L
GOT (AST)	30.00	0 – 35 U/L
Total Bilirubin	1.3	0.3 – 1.2 mg/dL
Gamma-GT	101.00	7 – 55 U/L
Total Cholesterol	86.00	100 – 200 mg/dL
Alkaline Phosphatase	163.00	42 – 128 U/L
INR	1.38	0.9 – 1.27

 At this point, the psychologist referred the patient to the imaging department, for a cerebral MRI to exclude other nervous system anomalies. The patient presented an otherwise normal clinical neurologic exam, with a normal EEG. 

**Fig. 1:**
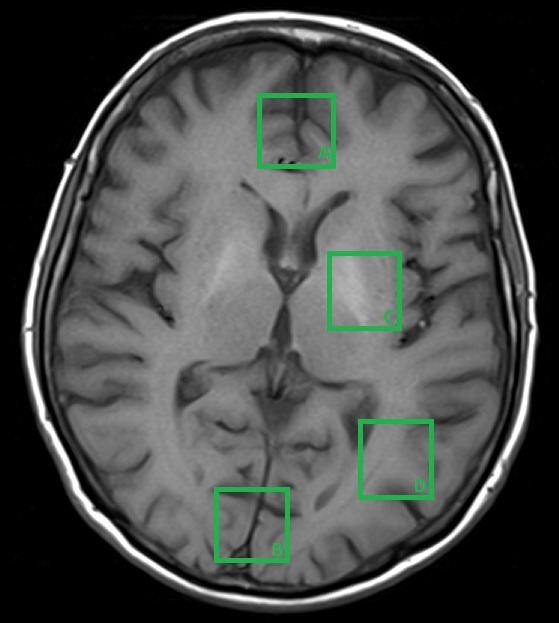
Common voxel locations for MRS spectrum, as follows: A. Bifrontal grey matter. B. Bilateral parieto-occipital grey matter. C. Basal ganglia. D. Parieto-occipital white matter.

 The radiologist on call carried out a routine MRI examination, and due to the diagnostic suspicion of MHE, he also performed a MRS sequence. The standard location of the voxel in the published studies in either frontal grey matter, basal ganglia or parietal white matter (**Fig. 1**). Some studies use the occipital grey matter for the voxel placement [**4**]. 

**Fig. 2:**
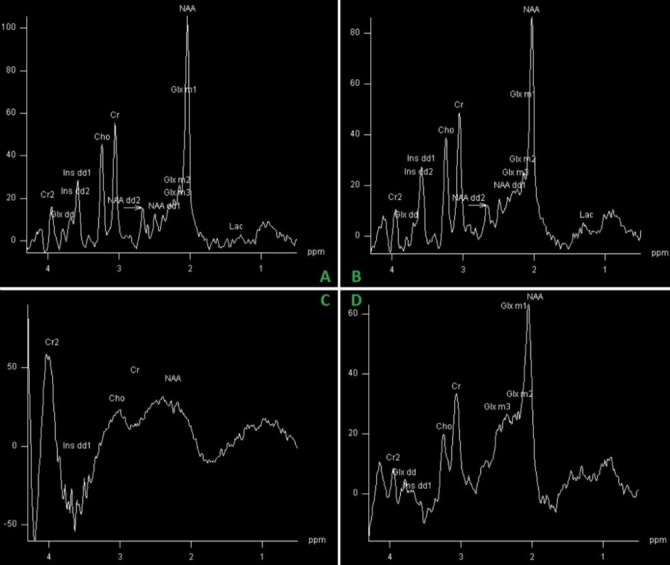
Spectra in the 4 locations depicted in **Fig. 1**. Spectra A and B are nearly identical and within normal range. Specter C is partly artifacted and inconclusive. Specter D is relevant for hepatic encephalopathy.

 Initial results did not yield any spectrum alterations, the results falling into standard limits. The volume of interest (VOI) had been placed in the bifrontal grey matter. The doctor insisted and employed other voxel locations, and in one case (parietal region), the spectrum showed conclusive evidence of hepatic encephalopathy (decreased choline and myo-inositol peaks and increased glutamate complex peak). The spectrum features hold virtually no differential diagnosis, being highly specific for this condition (**Fig. 2**). Correlated with the clinical interview of the patient, the final diagnosis of minimal hepatic encephalopathy was set. 

 The patient was initiated on treatment with probiotics and lactulose and showed a definite clinical improvement of the psychological status. The increased quality of life, along with the psychological counseling allowed the patient to cut off alcohol consumption and lose some of the excessive weight, nearing normal body mass index ranges. Medication corrected the gastrointestinal symptoms.

 The patient returned within 6 months for the surgical procedure, which took place without complications.

## Discussion

The diagnosis immediately followed by treatment of minimal hepatic encephalopathy significantly increases the quality of life of the patients, and decreases the risk of encephalopathy episodes and also the chance of evolution to clinical HE [**5**].

Increasing the quality of life of chronically ill patients augments medication compliance and helps patients in long-term health plans such as losing weight and following a healthy medically advised routine.

 Currently there are no validated tests for the diagnosis of minimal hepatic encephalopathy, the suspicion being raised on behavioral modifications detected by the patient or its entourage, and supported by altered neuropsychological tests.

 In the situation of an equivocal neuropsychological test, MRS can prove very useful in establishing the diagnosis.

 Although the physiopathological mechanisms of the onset and evolution of HE are complex [**6**], the MRS appearance is identical despite of the precipitating cause, and the magnetic resonance spectrum findings strongly support the diagnosis in this case.

*ACKNOWLEDGEMENT: This paper is supported by the Sectoral Operational Programme Human Resources Development (SOP HRD) 2007-2013, financed from the European Social Fund and by the Romanian Government under the contract number POSDRU/107/1.5/S/82839*
